# Smart green spectrophotometric assay of the ternary mixture of drotaverine, caffeine and paracetamol in their pharmaceutical dosage form

**DOI:** 10.1186/s13065-023-01097-9

**Published:** 2023-12-13

**Authors:** Rana Ghonim, Manar M. Tolba, Fawzia Ibrahim, Mohamed I. El-Awady

**Affiliations:** 1 Department of Pharmaceutical Analytical Chemistry, Faculty of Pharmacy, Mansoura University, Mansoura, 35516 Egypt; 2 Department of Pharmaceutical Chemistry, Faculty of Pharmacy, Delta University for Science and Technology, International Coastal Road, Gamasa, 11152 Egypt

**Keywords:** Petro, Drotaverine, Caffeine, Paracetamol, Derivative spectrophotometry, Double divisor, Mean centering

## Abstract

Three green and facile spectrophotometric methods were developed for the assay of Petro^®^ components; drotaverine HCl (DRT), caffeine (CAFF), and paracetamol (PAR). The three methods depend on measuring the absorbance of the studied drugs through their ethanolic solution. The first derivative spectrophotometry (FDS) at (Δλ = 10) were good parameters for DRT and CAFF resolution; DRT and CAFF could be well calibrated using FDS at 320 and 285 nm, respectively. PAR could be estimated at 308 nm utilizing the second derivative spectrophotometry (SDS). Method II relies on the double divisor ratio derivative spectroscopy (DDRDS). The first derivative was applied on each drug where they would be assayed at 309, 288, and 255 nm for DRT, CAFF, and PAR, respectively. Method III depends on the mean centering (MCR) technique. DRT, CAFF, and PAR could be determined at 309, 214, and 248 nm, respectively. The concentrations were rectilinear in the ranges of 2–20 µg/mL for DRT, 1.5–15 µg/mL for CAFF, and 2–40 µg/mL for PAR in double devisor and mean centering but PAR from 5 to 40 µg/mL in derivative method. Method validation was performed according to ICH guidelines assured by the agreement with the comparison method. In addition, greenness assessment of the proposed methods was investigated. The application of the proposed method was extended to analyse tablet dosage form and performing invitro dissolution testing.

## Introduction

Ultraviolet spectrophotometry is among the most convenient and useful quantitative and qualitative methods, especially in multicomponent analysis [1] by minimizing the cumbersome task of separating interferents [2]. Derivative spectrophotometry is a useful analytical method For gathering variable data from the spectra of overlapped bands and minimizing the effects of baseline shifts and tilts. It entails computing and plotting one of the spectral curves’ and mathematical derivatives.Consequently, a spectrum’s information content is presented in a manner that may be more useful [3]. The basis of double divisor ratio derivative spectroscopy (DDRDS) is the derivative of the ratio spectrum, which is produced by dividing the ternary mixture's absorption spectra by a standard spectrum of a mixture of two of the three compunds in the desired mixture. Utilizing the calibration graphs for each compound, which are obtained by measuring the amplitude chosen, the concentrations of the three compounds in their mixture are identified [4]. One of the newest and most effective spectrophotometric techniques for quantitative analysis of multicomponent mixtures, mean centering of ratio spectra (MCR), does not require any derivatization stages. Compared to chromatography, this procedure is recognized to be more efficient in terms of time and cost [5].

Drotaverine HCl (DRT), Caffeine (CAFF) and Paracetamol (PAR) are official in British pharmacopeia [7] and United States pharmacopeia [8]. Drotaverine HCl (DRT) is 1-[(3,4-diethoxyphenyl)methylene]-6,7-diethoxy-1,2,3,4-tetrahydroisoquinoline hydrochloride [7]. DRT is antispasmodic drug [9]. Numerous analytical methods assessed DRT in different matrices. Recent articles regarding DRT analysis like UV visible spectrophotometry [10–13], spectrofluorimetric methods [14], electrochemical methods [15–18], potentiometric titration methods [19], chromatographic methods [20–33]. Caffeine (CAFF) is 1,3,7-trimethyl 3,7 dihydro-1 h- purine 2,6-dione [7]. The advantages include a decrease in tiredness and weariness and an improvement in mood. It has various pharmacological effects, including raising gastric output, fostering lipolysis, boosting skeletal and muscular contraction, and raising diuresis. Long-term sleeplessness, peptic ulcers, and elevated serum cholesterol are the main negative consequences of caffeine [34]. Numerous analytical methods were used for the assessment of CAFF in variable matrices like UV/Visible spectrophotometric methods like [35–39], partial least‐squares algorithm (PLS) [40], chromatographic methods [41–52] and electrochemical methods [53–56]. Paracetamol (PAR) is N-(4-hydroxyphenyl) acetamide [6]. PAR has analgesic and antipyretic properties and anti-inflammatory activity [9]. Numerous analytical methods were used for the assessment of PAR in various matrices like UV/Visible spectrophotometric methods [57–61], spectrofluorimetric methods [62], electrochemical methods [63–69], chromatographic methods [70–74] and other methods like flow injection analysis [75], coupling of sequential injection analysis (SIA) and fluorometric solid phase transduction [76] and capillary zone electrophoresis [77]. The concurrent estimation of the studied medications was achieved by using spectrophotometric and HPTLC methods [33], sweeping-micellar electrokinetic chromatography [78], and RP-HPLC method [79].

DRT, CAFF, and PAR are co-formulated in one tablet dosage form under trade names Petro^®^ tablets containing 40 mg DRT, 60 mg CAFF, and 400 mg PAR. The pharmaceutical ratio of co-formulations was found to be 1:1.5:10 (w/w) for DRT: CAFF: PAR, respectively.

This work aims to represent new univariate spectrophotometric methods to evaluate the assay of DRT, CAFF, and PAR co-formulated in Petro^®^ tablets in a pharmaceutical ratio 1:1.5:10 (w/w).

## Experimental

### Apparatus and software

- UV-PC spectrophotometer (Shimadzu 1650), supplied with 1.0 cm quartz cells.
- An ultrasonic bath (model SS 101 H 230, USA) was used for sonication.
- Matlab R2022, an (8.2.0.701) software, was used for performing the wholly chemometric procedures. PLS Toolbox software, version 2.1, was used to carry out mean centering through our own written codes in Matlab software. MCR was performed using PLS toolbox software version 2.1.

### Materials and solvents

- Reference standard samples of DRT, CAFF, and PAR were purchased from Amoun Pharmaceutical Company in El-Obour City, Egypt. These samples were confirmed to have purity levels of 99.5, 99.5, and 99.4%, respectively.
- Petro^®^ tablets; 40 DRT, 60 CAFF, and 400 mg PAR per tablet, a product of Alphamoun Pharmaceuticals Co., industrial zone, Badr City, Egypt (batch No. 12101299), purchased from a local pharmacy in Egypt.
- The inactive ingredients other than DRT, CAFF and PAR in Petro tablet (Palcebo): magnesium stearate, lactose monohydrate, maize starch, calcium hydrogen phosphate dihydrate and talc were obtained from pharmaceutical chemistry department,faculty of pharmacy, delta university for science and technology.
- Filtered deionized water was used throughout the work.
- Ethanol, methanol, acetonitrile, propanol, and acetone were HPLC grade, were obtained from Fisher, UK.

### Preparation of standard solution

By dissolving 0.01 g of each drug in 100 mL of ethanol, standard stock solutions containing (100 µg/mL) of each investigated medicines were created. The working solutions were produced from the standard stock solutions by employing the serial dilution procedure with the same solvent for DRT, CAFF, and PAR.

### Procedures

#### Spectral features

From 200 to 400 nm, the absorption spectra of ethanolic solutions containing various DRT, CAFF, and PAR concentrations were scanned.

#### Calibration graphs development

–Derivative method

Accurately measured volumes of DRT, CAFF and PAR standard solutions were transferred into separate sets of 10 mL volumetric flasks to get concentrations in the range of 2–20 µg/mL for DRT, 1.5–15 µg/mL for CAFF, and 5–40 µg/mL for PAR in derivative method while 5–40 µg/mL, completed with ethanol to the mark. The absorption spectra of the prepared DRT, CAFF, and PAR solutions were recorded against ethanol as blank over 200–400 nm. The first derivative was then manipulated using scaling factor = 10.0, smoothing level (× 10), and Δλ = 10.0 nm. The trough amplitude was measured at 320 nm for DRT and 285 nm for CAFF. The SDS with Δλ = 10.0 nm, smoothing level (× 10), and scaling factor 20 is the optimum solution for PAR resolution at 307 nm. All drugs were measured and plotted against final concentration in μg/mL to develop a calibration graph. Alternatively, the regression equation was derived.

–Double divisor ratio derivative method

The ratio spectra were generated by recording the absorption spectra of the solutions produced at divergent concentrations of one of the drugs (DRT, CAFF, and PAR) and dividing them by the sum of the absorption spectra of the two other drugs. The ratio spectra of DRT were achieved by dividing DRT spectra against 9 µg/mL of CAFF and 8 µg/mL of PAR, called the double divisor for the ratio spectra of CAFF. CAFF absorption spectra were divided over 8 µg/mL DRT and 8 µg/mL PAR. Finally, For PAR ratio spectra, dividing PAR absorption spectra 6 µg/mL CAFF and 6 µg/mL DRT, the D^1^ of the ratio spectra were displayed with smoothing level(× 10) and scaling factor 10.

The concentrations of the studied drugs were estimated by measuring the amplitude at 309 nm for DRT, 288 nm for CAFF, and 255 nm for PAR and at, which matched the first derivative of the ratio spectra in the specified spectral region (200–400 nm). The amplitudes were measured and plotted against the final concentration in μg/mL to establish a calibration graph. Alternatively, the regression equation was derived.

–Mean Centering Method

For DRT: the recorded spectra were divided by the standard spectrum of 9 μg/mL CAFF and 8 μg/mL PAR to obtain the ratio spectra, which was then mean centered. Then the MCR was then obtained.

For CAFF, the recorded spectra were divided by 8 μg/mL DRT and 8 μg/mL PAR to obtain the first ratio spectra, which was then mean centered.

Similarly, the recorded spectra of PAR were divided by 6 μg/ml DRT and 6 μg/mL CAFF and the obtained ratio spectra were mean-centered.

The mean-centered values of the ratio spectra at 309, 214, and 248 nm for DRT, CAFF, and PAR, respectively, were measured and plotted against the corresponding concentration of each drug to construct their respective calibration graphs, then the regression equations were derived.

#### Determination of the studied drugs in synthetic mixtures

To generate a synthetic mixture of three variable concentrations within the required range, exact amounts of the working stock solutions of DRT, CAFF, and PAR were placed into 10 mL volumetric flasks.

#### Preparation of dosage form solutions (Petro^®^ tablets)

Ten tablets were triturated and weighed accurately. One tablet containing 40 mg DRT, 60 mg CAFF, and 400 mg PAR included an exact weight of powder extracted with a specific amount of ethanol, sonicated for 30 min, finished to the correct weight with ethanol, and then filtered. More adequate dilutions were made to prepare the samples within the drugs’ concentration range.

## Results and discussion

**Fig. 1:**
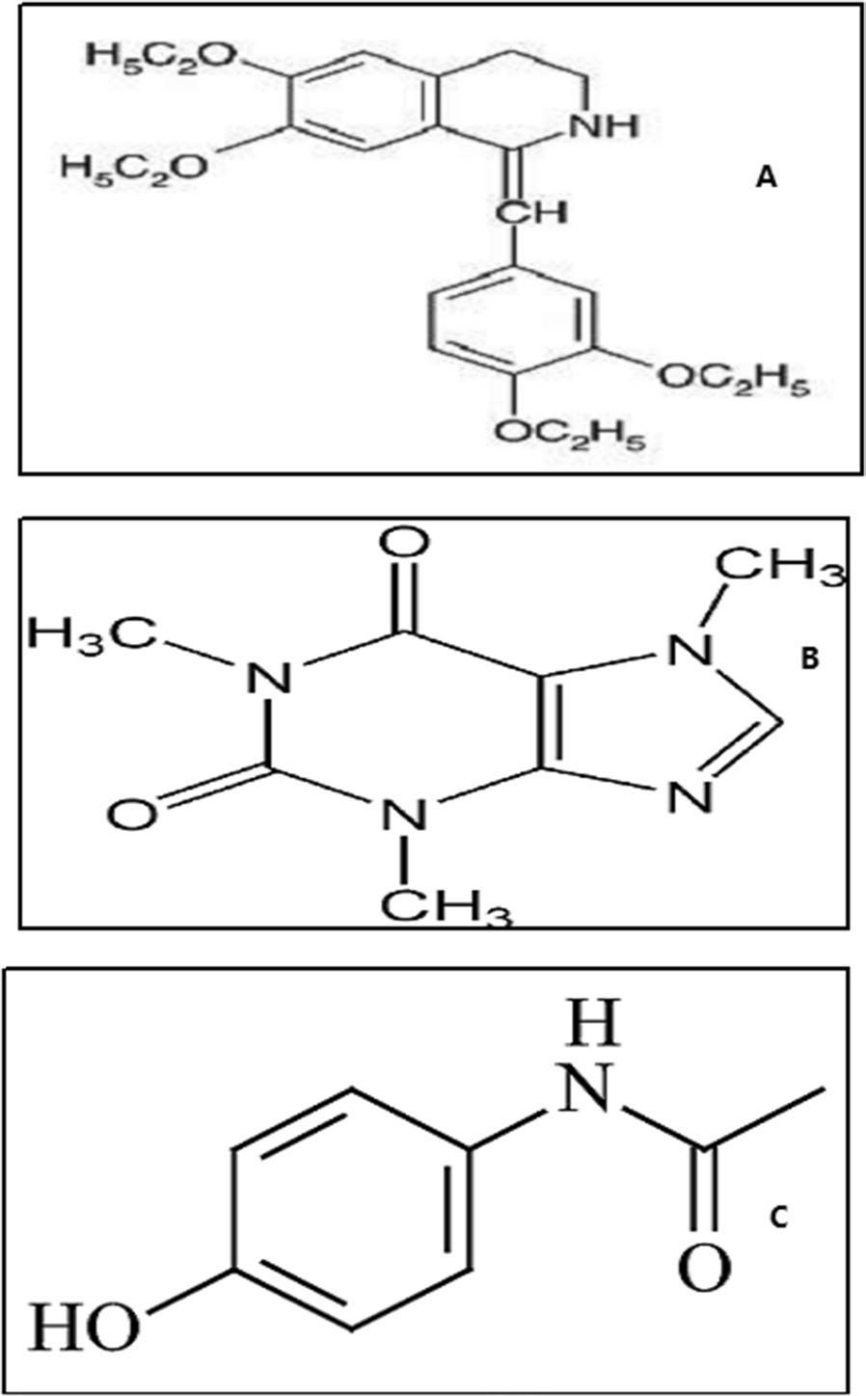
Chemical formulae of: (**A**) Drotaverine HCl, (**B**) Caffeine, (**C**) Paracetamol

**Fig. 2:**
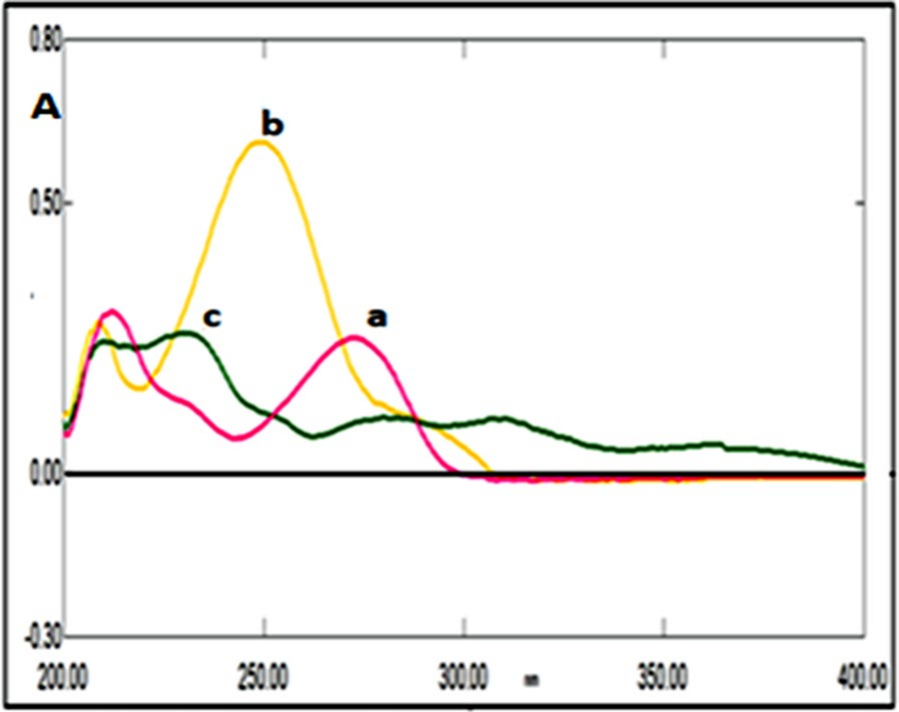
Zero order absorption spectra of DRT, CAFF, and PAR ethanolic solution: (**a**) DRT [8.0 µg/mL], (**b**) CAFF (8.0 µg/mL), (**c**) PAR (8.0 µg/mL)

This study set out to estimate DRT, CAFF, and PAR (Fig. 1) in their ternary mixture using straightforward univariate methods. Since their UV-absorption spectra had a lot of overlap, as seen in Fig. 2, it was difficult to determine them directly. While the spectra of DRT, CAFF, and PAR could be easily resolved and calculated upon applying the proposed approaches.

### Derivative technique (Method I)

**Fig. 3:**
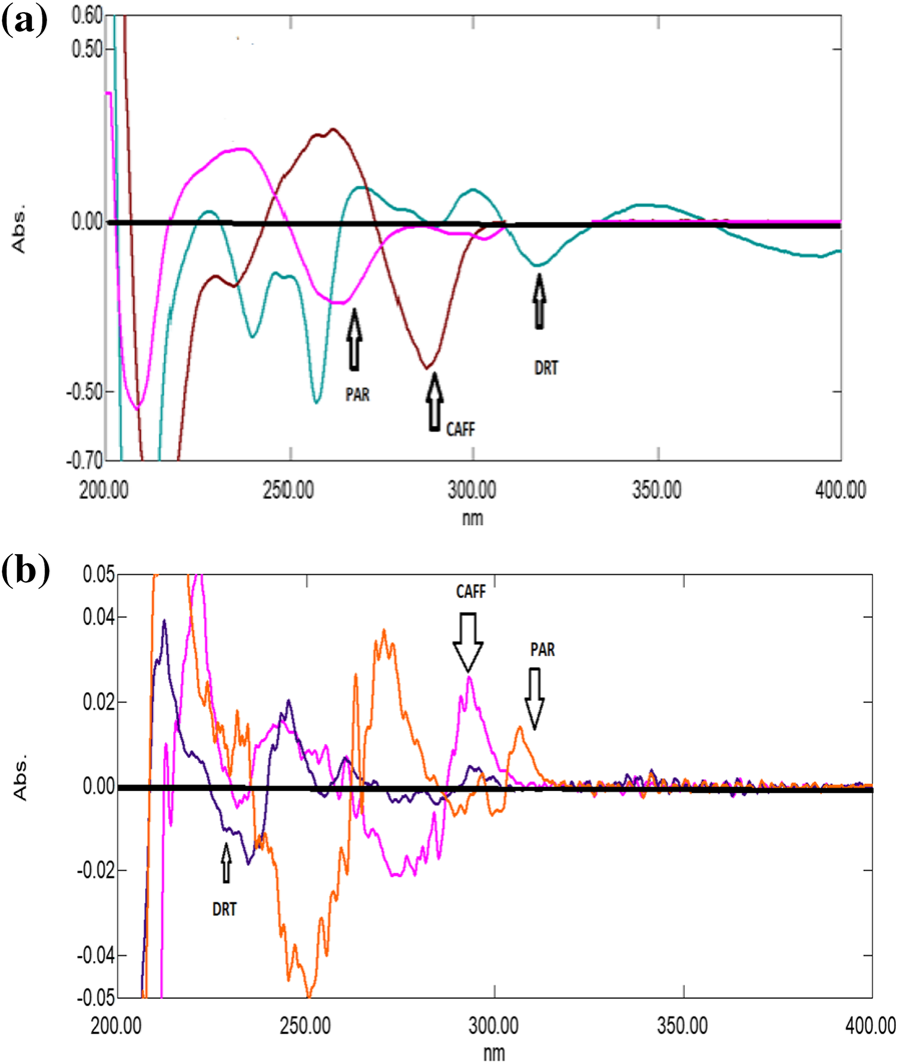
**a**: First derivative spectrophotometry for 10 µg/mL DRT, 6 µg/mL CAFF and 15 µg/mL PAR. **b**: Second derivative spectrophotometry for 10 µg/mL DRT, 6 µg/mL CAFF and 15 µg/mL PAR

**Fig. 4:**
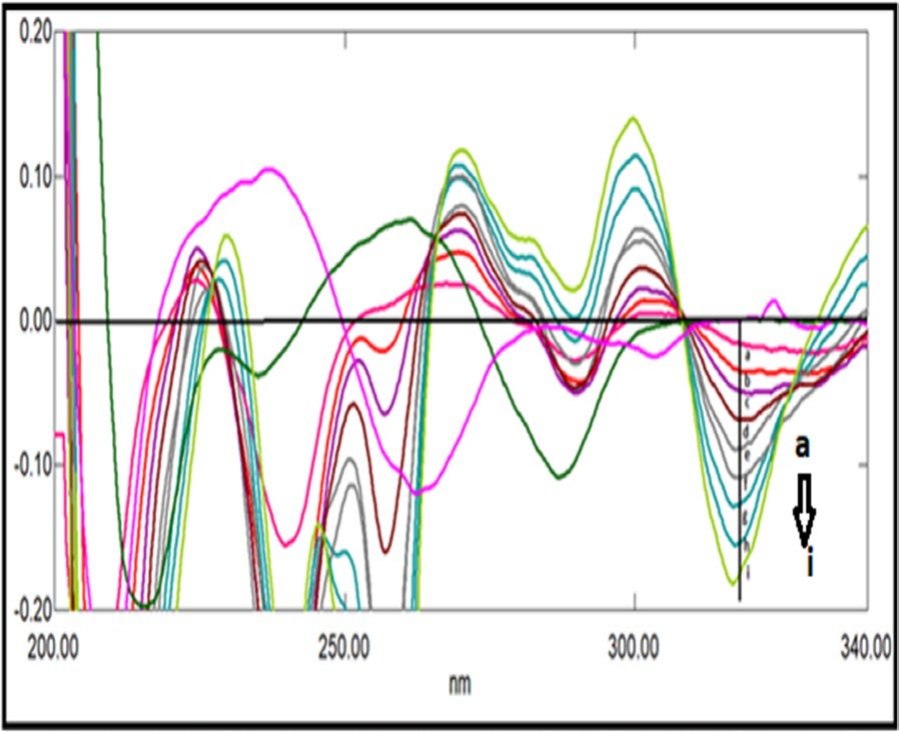
First derivative spectrophotometry for different concentrations of DRT from 2 to 20 µg/mL at 309 nm

**Fig. 5:**
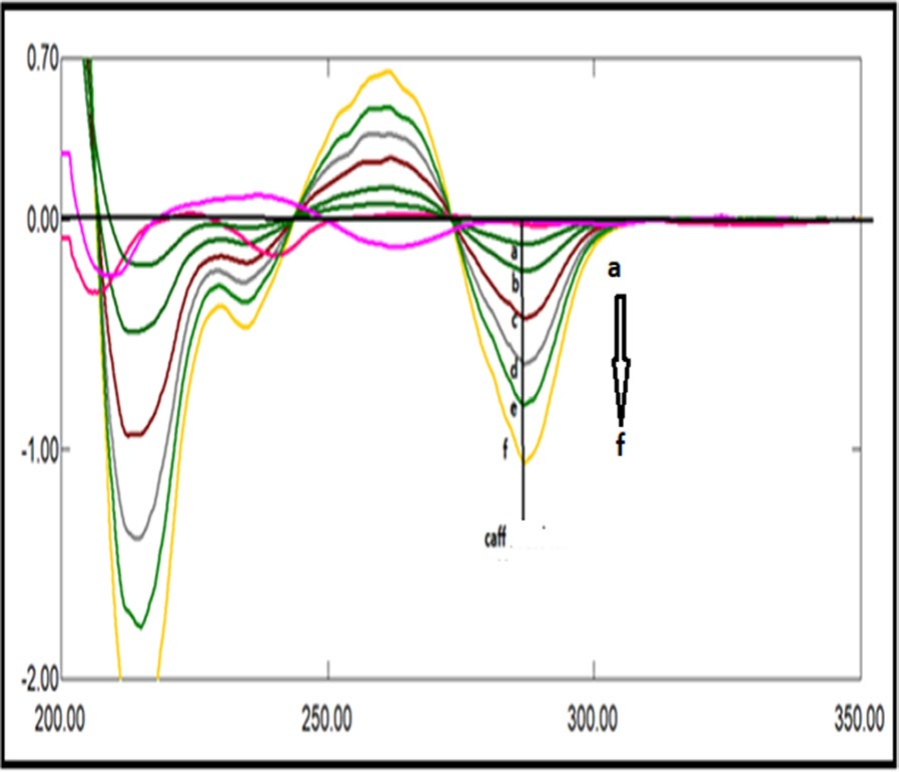
First derivative spectrophotometry for different concentrations of CAFF from 1.5 to 15 µg/mL at 285 nm

**Fig. 6:**
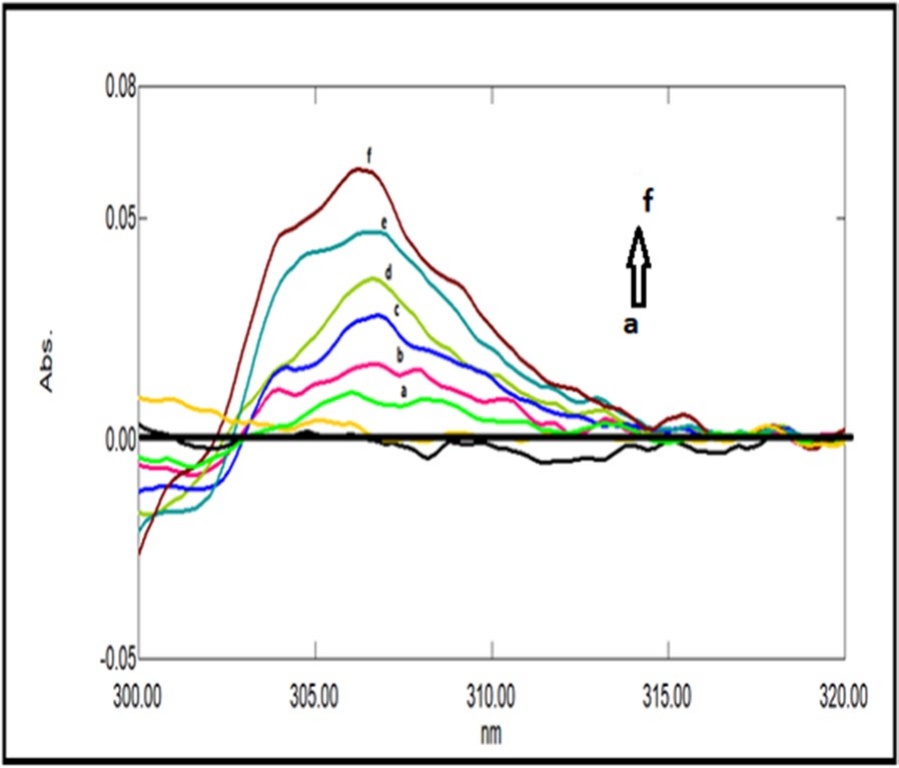
Second derivative spectrophotometry for different concentrations of PAR from 5 to 40 µg/mL at 307 nm

A great overlapping was observed between the absorption spectra of DRT, CAFF, and PAR, which is absurd to be separated by conventional spectrophotometry (Fig. 2). So, the derivative technique is a good alternative for improving the selectivity. Divergent smoothing levels and scaling factor values were examined to enhance this mixture's resolution. It was found that first derivative spectrophotometry with scaling factor 20, Δ λ = 10, and smoothing level 10 were good parameters for DRT and CAFF resolution (Fig. 3a). DRT and CAFF first derivative absorption spectra were resolved, while for PAR resolution and separation; the second derivative with Δλ = 10.0 nm smoothing level (× 10) and scaling factor 20 was applied (Fig. 3b). DRT and CAFF could be well calibrated using FDS at 320 and 285 nm, respectively (Figs. 4, 5). PAR could be well calibrated using Second derivative spectrophotometry (SDS) at 307 nm (Fig. 6). These wavelengths were chosen as they are zero crossing points for the other drugs and have accurate and reproducible results.

### Double divisor technique (Method II)

**Fig. 7:**
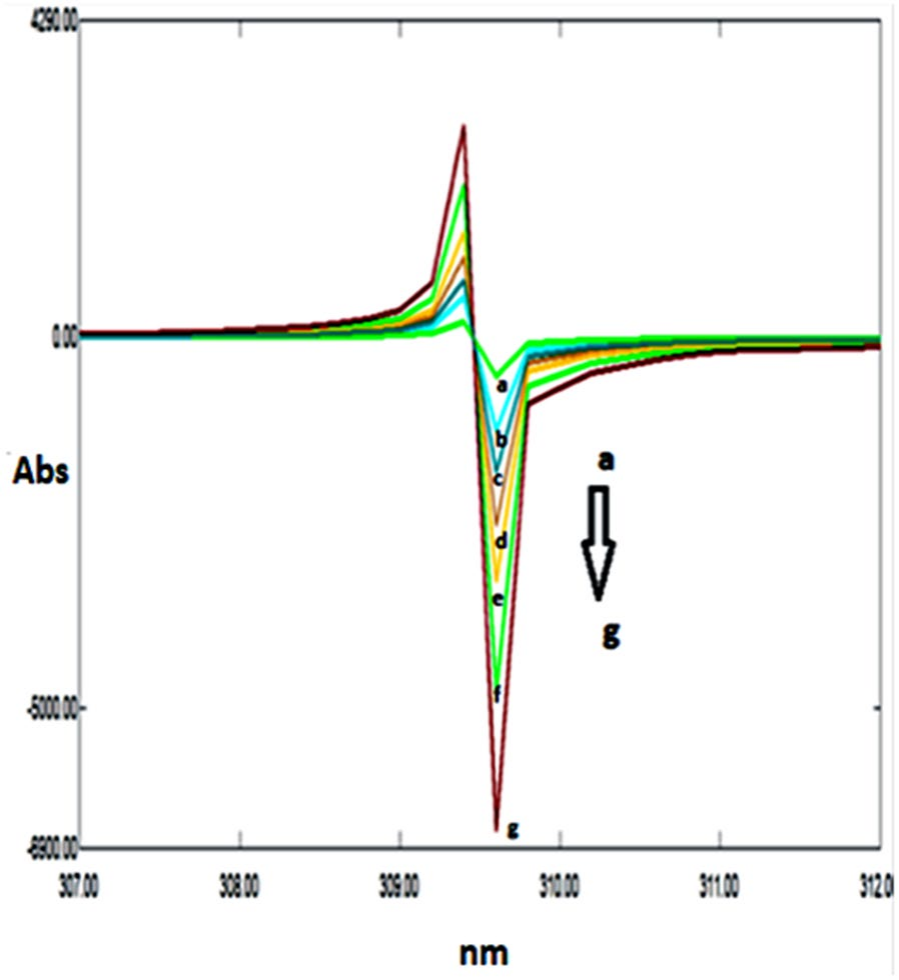
Different absorption ratio spectra of DRT divided by the double divisor (9 μg/mL CAFF + 8 μg/mL PAR)

**Fig. 8:**
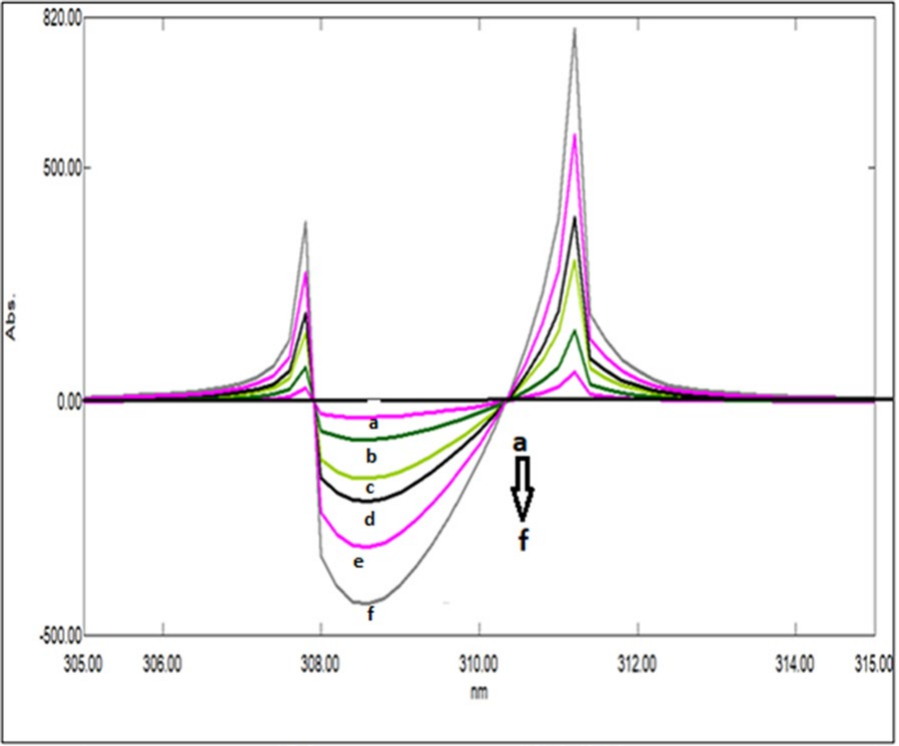
Different concentrations of first derivative ratio spectra of DRT at 309.0 nm

Figure 7 illustrates the ratio spectra of variable DRT concentrations and by applying the first derivative of the ratio spectra was obtained by utilizing a Δλ = 10, smoothing level (× 10), and a scaling factor of 10, then reproducible peaks were selected from the resulting derivative ratio spectra to estimate DRT. DRT amplitudes were calculated at 309.0 nm, as shown in Fig. 8.

**Fig. 9:**
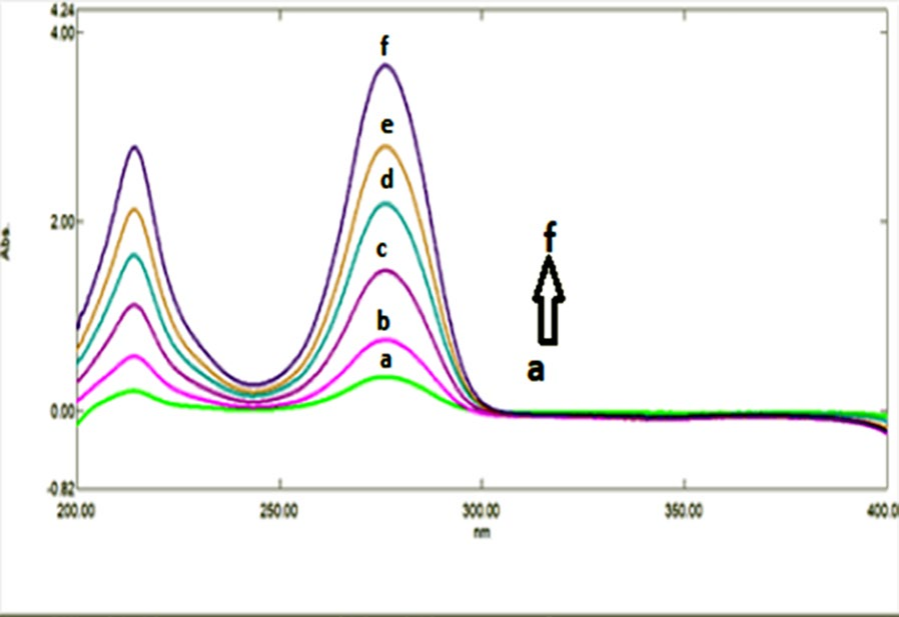
Different absorption ratio spectra of CAFF divided by the double divisor (8 μg/mL DRT + 8 μg/mL PAR)

**Fig. 10:**
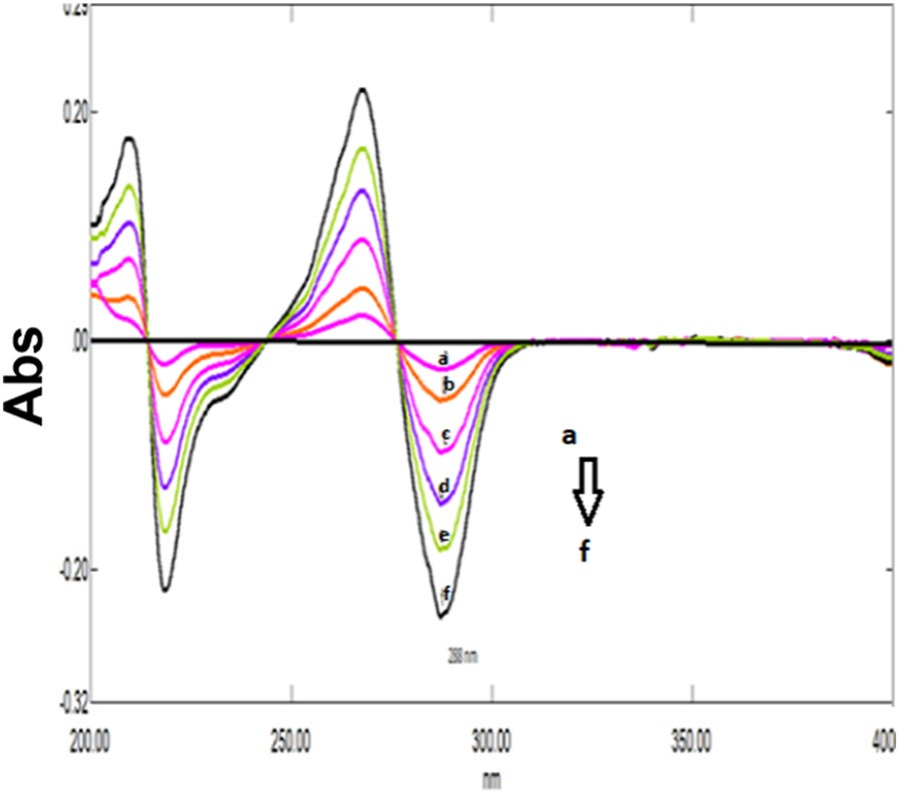
Different concentrations of first derivative ratio spectra of CAFF at 288 nm

Figure 9 illustrates the ratio spectra of variable CAFF concentrations by applying the first derivative of the ratio spectra was obtained by utilizing a Δ λ = 10, smoothing level (× 10), and a scaling factor of 10. In Fig. 10, reproducible peaks were selected from the resulting derivative ratio spectra to estimate CAFF at 285.

**Fig. 11:**
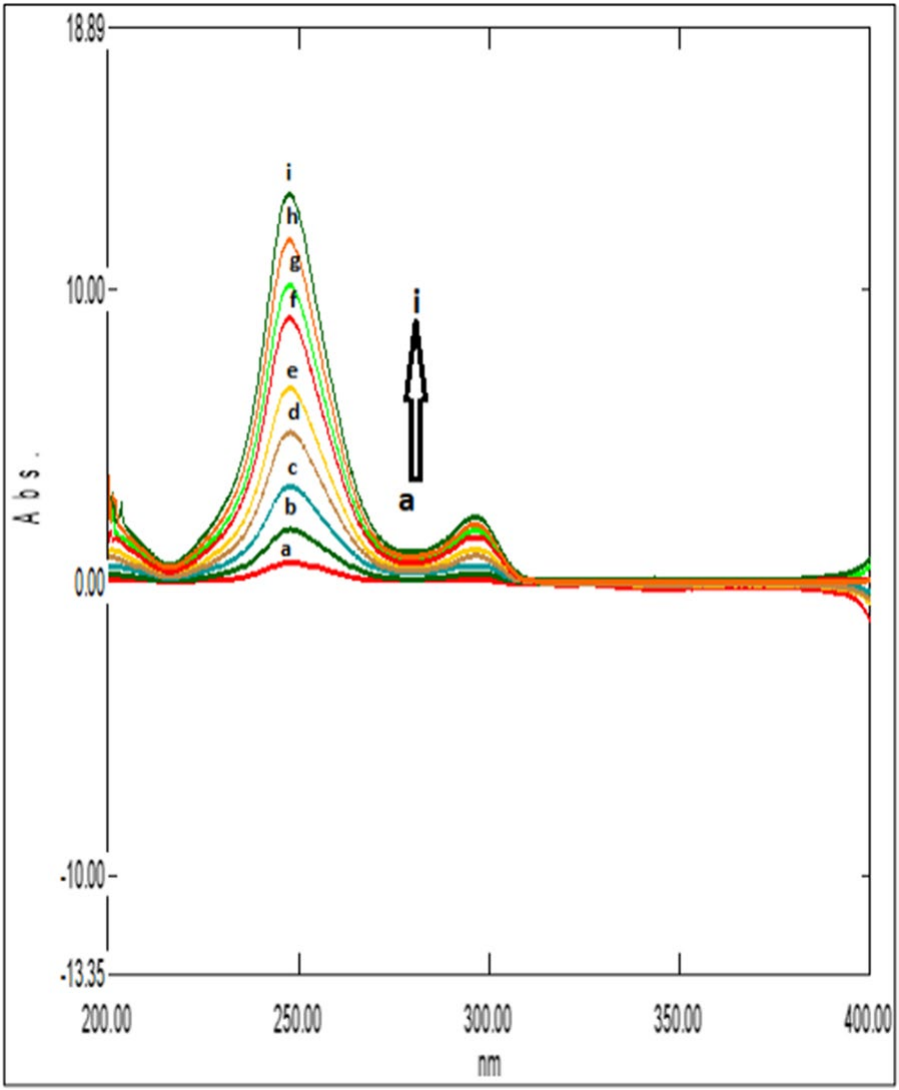
Different absorption ratio spectra of PAR divided by the double divisor (6 μg/mL DRT + 6 μg/mL CAFF)

**Fig. 12:**
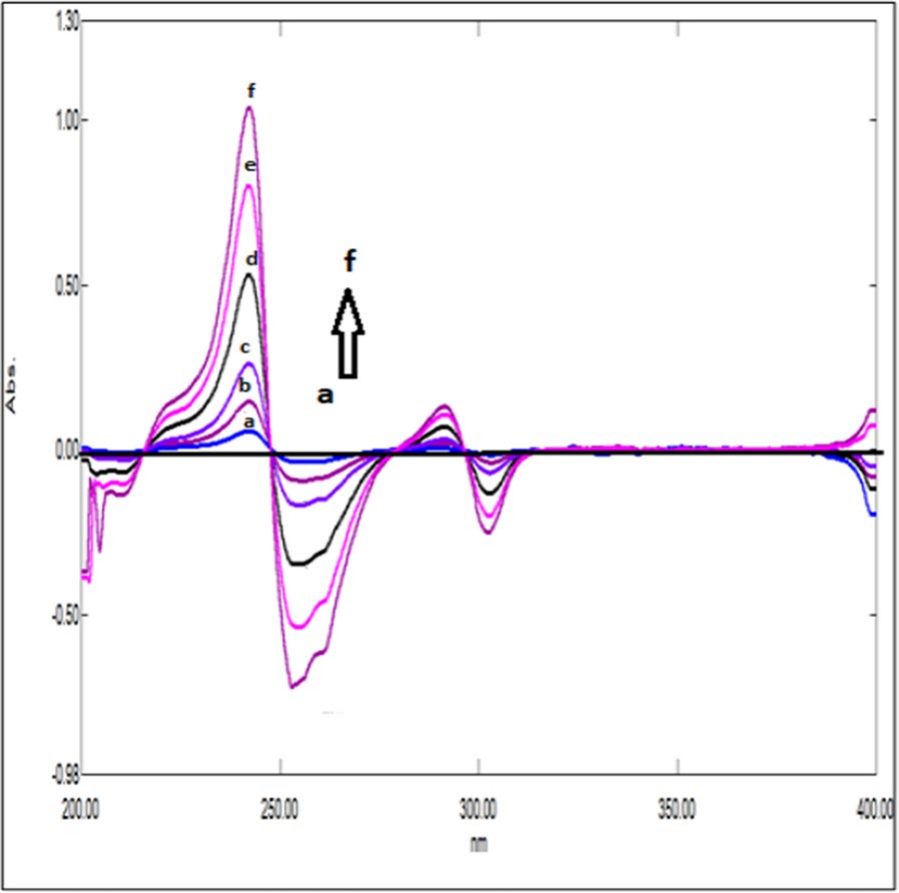
Different concentrations of first derivative ratio spectra of PAR at 248 nm

Figure 11 illustrates the ratio spectra of variable PAR concentrations. The first derivative spectra of the ratio spectra were obtained by a Δλ = 10, smoothing level (× 10), and a scaling factor of 10. Reproducible peaks were selected from the resulting derivative ratio spectra to determine PAR at 255.0 nm, as shown in Fig. 12.

The amplitudes of DRT, CAFF and PAR at the selected wavelengths were plotted against the corresponding drug concentrations.

### Mean centering of ratio spectra spectrophotometric method (MCR) method (Method III)

**Fig. 13:**
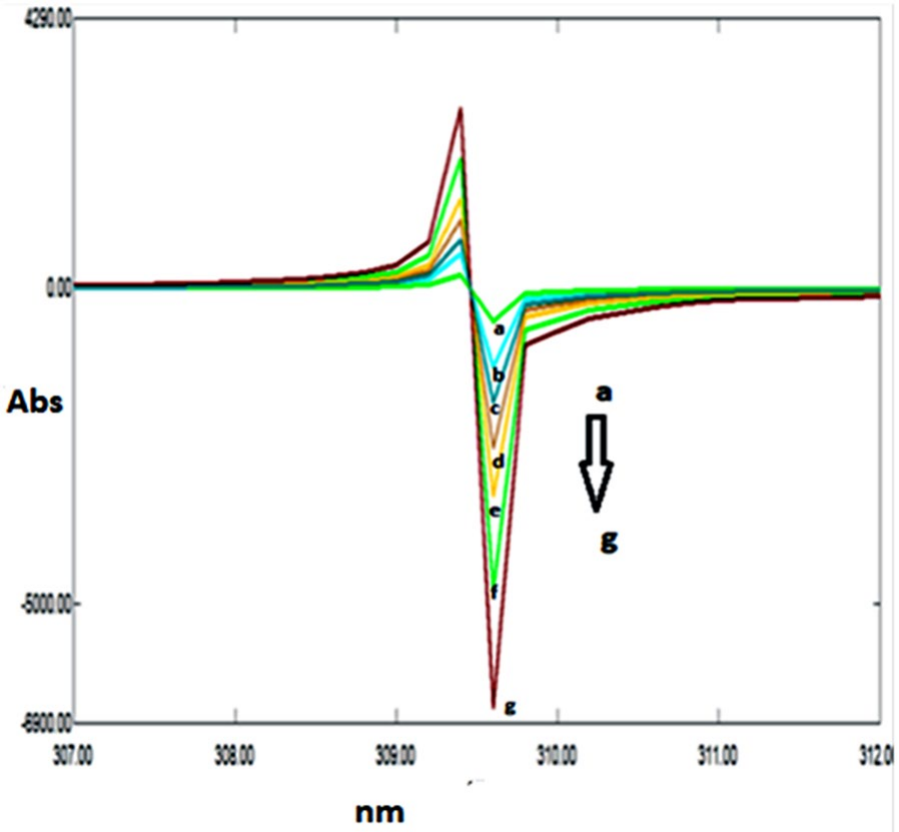
Calibration curves relating the mean-centered values at 309 nm to the corresponding concentrations of DRT

**Fig. 14:**
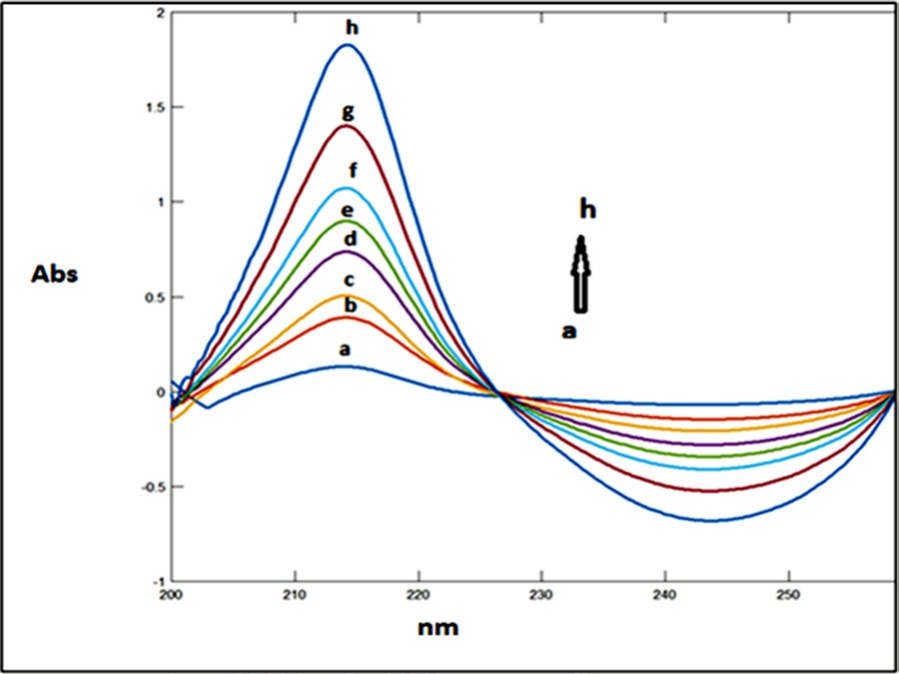
Calibration curves relating the mean-centered values at 214 nm to the corresponding concentrations of CAFF

**Fig. 15:**
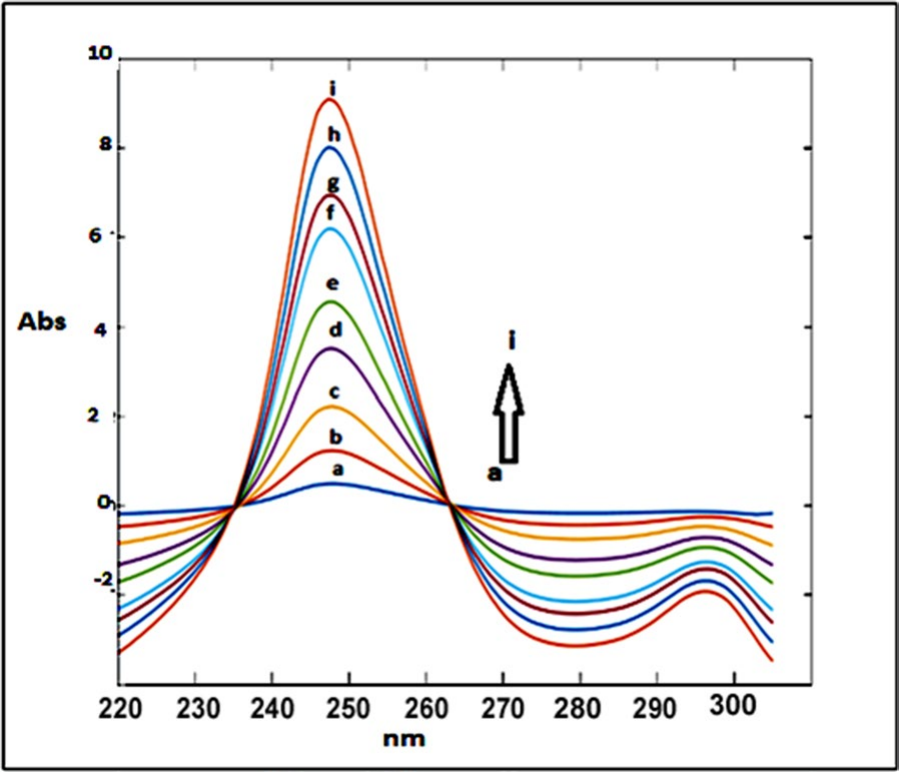
Calibration curves relating the mean-centered values at 248 nm to the corresponding concentrations of PAR

After studying these parameters, it was found that the divisor had a great effect on the selectivity of determination of the studied drugs where reproducible and good results were obtained upon using concentrations of 9 μg/mL and 8 μg/mL each of CAFF and PAR (for DRT) and 8 μg/mL and 8 μg/mL each of DRT and PAR (for CAFF) and 6 μg/mL each of CAFF and DRT (for PAR) as divisors. Figures 13, 14, 15 are the calibration curves relating the mean-centered values at 309, 214, and 248 nm to the corresponding concentrations of DRT, CAFF, and PAR, respectively, have been constructed from which the regression equation parameters.

#### Methods optimization

–Effect of diluting solvent

**Fig. 16:**
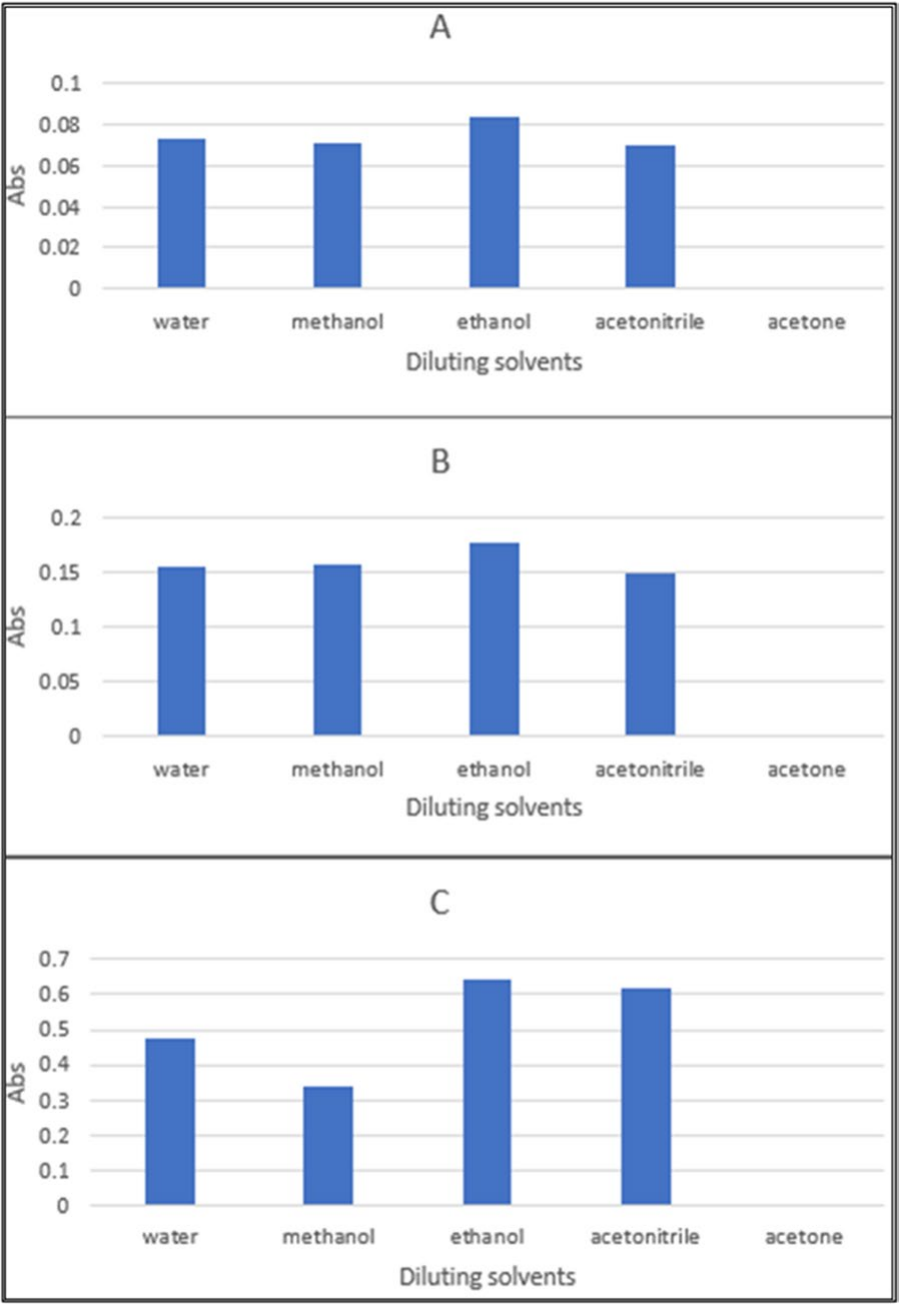
Effect of diluting solvents on 10 µg/mL of each: (**A**) DRT, (**B**) CAFF, (**C**) PAR

Different diluting solvents were examined, like distilled water, methanol, ethanol, acetonitrile, and acetone, and it was found that ethanol is the best diluting solvent for the studied drugs as it gave high absorbance (Fig. 16).

- The optimization of the derivative spectrophotometric method scanned by different values of Δλ, smoothing level and scaling factor were examined to improve resolution of this mixture. It is found that first derivative spectrophotometry with Δλ = 10, scaling factor 20 and smoothing level 10 were good parameters for DRT and CAFF resolution while for PAR resolution is second derivative scaling factor 20 smoothing level × 10.
- The optimization of the double divisor ratio derivative come by changing the concentrations of the double divisor till reaching the best concentration for accuracy and reproducibility. The double divisor in case of DRT is 9 μg/mL CAFF and 8 μg/mL PAR, CAFF is 8 μg/mL DRT + 8 μg/mL PAR and PAR is 6 μg/mL CAFF + 6 μg/ml DRT. Changing the Δλ from 10 to 40 nm till found 10 nm is the best, smoothing level until 10 and also scaling factor was scanned like 1, 5, 10 and 20 till found that 10 is the best.
- The optimization in the mean centering method determined through the choice of the divisor and hence the vectors that selected for mean centering and the range of the spectrum. After studying these parameters, it was found that 200–400 nm is the UV spectrophotometric range. The divisor had a great effect on the selectivity of determination of DRT, CAFF and PAR where reproducible and good results have been obtained upon using concentration of 9 μg/mL and 8 μg/mL each of CAFF and PAR (for DRT) and 8 μg/mL and 8 μg mL^−1^ each of DRT and PAR (for CAFF) and 6 μg/mL each of CAFF and DRT (for PAR) as divisors. DRT, CAFF and PAR could be determined at 309, 214 and 248 nm, respectively.

#### Method validation

The validation parameters were studied according to ICH recommendations [80].

The linearity of the proposed methods was estimated through the calibration graphs by plotting the amplitude of the first derivative *versus* the concentration of DRT, CAFF, and PAR at 320.0, 285, and 307 nm, respectively, in the case of the derivative method and DRT, CAFF, and PAR at 309, 288, and 255 nm respectively in case of double divisor method.

The linearity of the mean centering was estimated through the calibration graphs by plotting absorbance response *versus* the concentration of DRT, CAFF, and PAR at 309.0 nm, 214.0 nm, and 248 nm, respectively, in the case of the mean centering method.

The concentrations were rectilinear in the ranges of 2–20 µg/mL for DRT, 1.5–15 µg/mL for CAFF, and 2–40 µg/mL for PAR in double devisor and mean centering but PAR from 5 to 40 µg / mL in derivative method.

High correlation coefficients (r) of the regression equations, small residual standard deviation (Sy/x) and percentage relative standard deviation (%RSD) values, intercept and slope standard deviation (Sa), are all produced through statistical analysis of the data (Tables 1, 2, 3). Limits of Quantitation and detection (QL) (DL) were calculated according to ICH Q2 (R1) recommendations [80] are also abridged in Tables 1, 2, and 3.

**Table 1:** Analytical performance data for the determination of DRT, CAFF and PAR by the derivative method

Parameter	DRT	CAFF	PAR
Wavelength (nm)	320 nm	285 nm	307 nm
Linearity range (µg/mL)	2–20	1.5–15	5–40
Intercept (*a*) × 10^–3^	− 3.7	0.0098	7.8
Slope (*b*) × 10^–3^	9	0.0691	0.8
Correlation coefficient (*r*)	0.9999	0.9998	0.9999
S.D. of residuals (S_*y/x*_) × 10^–3^	0.70	5.80	0.420
S.D. of intercept (S_*a*_) × 10^–3^	0.40	3.40	0.33
S.D. of slope (S_*b*_) × 10^–3^	0.05	0.05	0.01
% RSD^a^	0.94	1.02	0.46
*%* Error^b^	0.35	0.42	0.19
LOD^c^ (µg/mL)	0.14	0.16	1.37
LOQ^d^ (µg/mL)	0.43	0.48	4.14

^a^Percentage relative standard deviation

^b^Percentage relative error

^c^Limits of detection

^d^Limits of quantitation

**Table 2:** Analytical performance data for the determination of DRT, CAFF and PAR by the double divisor method

Parameter	DRT	CAFF	PAR
Wavelength (nm)	309 nm	288 nm	255 nm
Linearity range (µg/mL)	2–20	1.5–15	2–40
Intercept (*a*) × 10^–3^	− 8.360	0.0002	0.0147
Slope (*b*) × 10^–3^	22.164	0.016	0.026
Correlation coefficient (*r*)	0.9998	0.9999	0.9999
S.D. of residuals (S_*y/x*_) × 10^–3^	2.72	0.001	0.004
S.D. of intercept (S_*a*_) × 10^–3^	1.46	0.7 × 10^–3^	2 × 10^–3^
S.D. of slope (S_*b*_) × 10^–3^	0.18	0.1 × 10^–3^	0.1 × 10^–3^
% RSD^a^	1.08	0.75	0.9
*%* Error^b^	0.44	0.31	0.37
LOD^c^ (µg/mL)	0.22	0.14	0.24
LOQ^d^ (µg/mL)	0.66	0.42	0.71

^a^Percentage relative standard deviation

^b^Percentage relative error

^c^Limits of detection

^d^Limits of quantitation

**Table 3:** Analytical performance data for the determination of DRT, CAFF and PAR by the mean centering method

Parameter	DRT	CAFF	PAR
Wavelength (nm)	309 nm	214 nm	248 nm
Linearity range (µg/mL)	2–20	1.5–15	2–40
Intercept (*a*) × 10^–3^	8.812	57.95	7.16
Slope (*b*) × 10^–3^	3.061	344.42	0.790
Correlation coefficient (*r*)	0.9999	0.9999	0.9999
S.D. of residuals (S_*y/x*_) × 10^–3^	0.34	26.01	0.28
S.D. of intercept (S_*a*_) × 10^–3^	0.24	11.81	0.16
S.D. of slope (S_*b*_) × 10^–3^	0.02	2.37	0.004
% RSD^a^	1.05	1.12	0.87
*%* Error^b^	0.43	0.46	0.36
LOD^c^ (µg/mL)	0.26	0.11	0.67
LOQ^d^ (µg/mL)	0.79	0.34	2.03

^a^Percentage relative standard deviation

^b^Percentage relative error

^c^Limits of detection

^d^Limits of quantitation

The repeatability of the method was determined by using three concentrations (4, 8, and 10 μg/mL) for DRT and (3, 6, and 15 μg/mL) for CAFF, and (10, 20 and 40 μg/mL) for PAR 3 times intra-daily and interday using the proposed univariate methods. Good results and acceptable % RSDs (less than 2%) were obtained, as summarized in Tables 4, 5, and 6.

**Table 4:** Precision data for determination of DRT, CAFF and PAR by the derivative method

Parameters		DRT concentration (μg/mL)			CAFF concentration (μg/mL)			PAR concentration (μg/mL)		
		**4.00**	**8.00**	**10.00**	**3.00**	**3.00**	**15.00**	**10.00**	**20.00**	**40.00**
Intra-day	Mean	100.58	99.47	100.05	100.64	99.5	100.05	100.06	99.90	99.98
	± SD	0.15	0.88	0.07	0.90	0.74	0.07	0.21	0.45	0.30
	% RSD	0.15	0.88	0.07	1.00	0.74	0.07	0.21	0.45	00.31
	% Error	0.51	0.51	0.04	0.58	0.43	0.04	0.12	0.03	0.04
Inter-day	Mean	100.0	100.34	99.61	100.25	99.90	99.61	98.18	100.5	99.95
	± SD	0.89	0.99	0.78	1.00	1.82	0.78	0.22	1.36	0.58
	% RSD	0.89	0.99	0.78	0.90	1.82	0.78	0.23	1.35	0.58
	% Error	0.087	0.58	0.46	0.52	1.06	0.46	0.14	0.78	0.18

**Table 5:** Precision data for determination of DRT, CAFF and PAR by the double divisor method

Parameters		DRT concentration (μg/mL)			CAFF concentration (μg/mL)			PAR concentration (μg/mL)		
		**4.00**	**8.00**	**10.00**	**3.00**	**6.00**	**15.00**	**10.00**	**20.00**	**40.00**
Intra-day	Mean	99.35	100.34	100.19	100.31	99.91	100.19	98.91	100.82	99.84
	± SD	0.48	0.25	0.17	1.06	0.55	0.17	0.96	0.49	0.05
	% RSD	0.48	0.25	0.17	1.06	0.55	0.17	0.97	0.49	0.05
	% Error	0.28	0.15	0.1	0.81	0.32	0.10	0.56	0.56	0.03
Inter-day	Mean	99.61	99.59	99.72	100.54	100.24	99.72	100.52	99.44	100.03
	± SD	1.13	1.06	0.56	1.39	0.63	0.56	0.98	0.97	0.24
	% RSD	1.14	1.07	0.56	1.39	0.63	0.56	0.98	0.97	0.24
	% Error	0.66	0.62	0.33	0.61	0.36	0.33	0.56	0.29	0.14

**Table 6:** Precision data for determination of DRT, CAFF and PAR by the mean centering method

Parameters		DRT concentration (μg/mL)			CAFF concentration (μg/mL)			PAR concentration (μg/mL)		
		**4.00**	**8.00**	**10.00**	**3.00**	**6.00**	**15.00**	**10.00**	**20.00**	**40.00**
Intra-day	Mean*	99.40	100.33	100.05	100.60	99.56	100.04	100.51	99.52	100.09
	± SD	1.07	0.33	0.08	1.00	0.52	0.08	0.52	0.49	0.10
	% RSD	1.08	0.32	0.08	1.00	0.52	0.08	0.52	0.49	0.10
	% Error	0.62	0.35	0.05	0.57	0.44	0.05	0.29	0.29	0.05
Inter-day	Mean*	99.25	100.68	99.63	99.13	100.74	99.63	100.12	99.48	99.84
	± SD	0.44	0.60	0.55	0.55	0.75	0.55	1.44	1.63	0.39
	% RSD	0.44	0.60	0.55	0.56	0.75	0.55	1.44	1.63	0.39
	% Error	0.25	0.19	0.32	0.32	0.30	0.32	0.83	0.94	0.23

^*^Each result was the average of three separate determinations

To evaluate the accuracy of the proposed methods for the studied drugs, whether alone or in their synthetic mixtures within their linearity ranges. The recovery percentages obtained are illustrated in Tables 7, 8, and 9 and Tables 10 and 11 for the synthetic mixtures. The suggested methods proved accurate, as revealed by the high recoveries values and low standard deviations. Statistical analysis of the results obtained by both the proposed and the comparison methods [79] was performed. F-test and t-test tested the difference between methods. The test ascertained no significant difference in accuracy and precision between the proposed and the comparison methods.

**Table 7:** Assay results for determination of DRT, CAFF and PAR in pure forms in the derivative method

Proposed method										Comparison method (79)					
Parameter	DRT			CAFF			PAR			DRT		CAFF		PAR	
	Amount taken µg/mL	Amount found µg/mL	% found^b^	Amount taken µg/mL	Amount found µg/mL	% found^b^	Amount Taken µg/mL	Amount Found µg/mL	% found^b^	Amount taken µg/mL	% found^b^	Amount Taken µg/mL	% found^b^	Amount taken µg/mL	% found^b^
	2	2.013	100.65	1.5	1.494	99.60	5	5.085	101.7	6.00	100.65	6.00	98.97	15.00	98.93
	4	3.940	98.50	3	3.040	101.33	10	10.127	101.27	8.00	98.60	8.00	98.29	20.00	101.90
	6	5.988	99.80	6	6.051	100.85	15	15.152	101.01	10.00	100.52	10.00	101.17	30.00	100.93
	8	7.921	99.01	9	8.919	99.10	20	20.168	100.84						
	10	9.966	99.66	12	11.871	98.93	30	30.189	100.63						
	14	14.166	101.19	15	15.125	100.83	40	40.704	101.76						
Mean			99.73			100.11			100.51						
± SD	0.93			1.02			1.08								
*t*^a^	0.66 (2.36)			0.75 (2.36)			0.09 (2.36)								
*F*^a^	1.12 (5.76)			2.84 (5.76)			1.39 (5.76)								

^a^The figures between parenthesis are the tabulated *t* and *F* values, respectively are at *P* = *0.05* [81]

^b^Each result was the average of three separate determinations

**Table 8:** Assay results for determination of DRT, CAFF and PAR in pure forms in the double divisor method:

Proposed method										Comparison method (79)					
Parameters	DRT			CAFF			PAR			DRT		CAFF		PAR	
	Amount taken µg/mL	Amount found µg/mL	% found ^b^	Amount taken µg/mL	Amount found µg/mL	% found^b^	Amount taken µg/mL	Amount found µg/mL	% found^b^	Amount taken µg/mL	% found^b^	Amount taken µg/mL	% found^b^	Amount taken µg/mL	% found^b^
	2	1.995	99.75	1.5	1.506	100.40	2	1.973	98.65	6.00	100.65	6.00	98.97	15.00	98.93
	4	3.942	98.55	3	3.025	100.83	5	4.94	98.80	8.00	98.60	8.00	98.29	20.00	101.90
	8	7.949	99.36	6	6.001	100.00	10	9.974	99.74	10.00	100.52	10.00	101.17	30.00	100.93
	10	10.009	100.09	9	8.912	99.02	20	20.087	100.44						
	14	14.210	101.72	12	11.886	99.05	30	30.28	100.93						
	20	19.85	99.30	15	15.051	100.34	40	39.78	99.45						
Mean			99.79			99.94			99.66						
± SD	1.07			0.75			0.89								
*t*^a^	0.87 (2.36)			0.64 (2.36)			1.17 (2.36)								
*F*^a^	3.52 (19)			13.15 (19)			2.84 (5.76)								

^a^The figures between parenthesis are the tabulated *t* and *F* values, respectively are at *P* = *0.05* [81]

^b^Each result was the average of three separate determinations

**Table 9:** Assay results for determination of DRT, CAFF and PAR in pure forms in the mean centering method

Proposed method										Comparison method (79)					
Parameters	DRT			CAFF			PAR			DRT		CAFF		PAR	
	Amount taken µg/mL	Amount found µg/mL	% found ^b^	Amount taken µg/mL	Amount found µg/mL	% found^b^	Amount taken µg/mL	Amount found µg/mL	% found^b^	Amount taken µg/mL	% found^b^	Amount taken µg/mL	% found^b^	Amount taken µg/mL	% found^b^
	2	2.026	101.3	1.5	1.515	101.00	2	1.996	99.80	6.00	100.65	6.00	98.97	15.00	98.93
	4	3.957	98.93	3	3.052	101.73	5	4.989	99.78	8.00	98.60	8.00	98.29	20.00	101.90
	6	5.989	9982	4.5	4.480	99.56	10	10.104	101.04	10.00	100.52	10.00	101.17	30.00	100.93
	8	7.918	98.98	6	6.017	100.28	20	19.696	98.48						
	10	10.126	101.26	9	8.866	98.51	30	30.327	101.09						
	20	19.981	99.91	15	15.068	100.45	40	98.867	99.67						
Mean			100.03			100.25			99.97						
± SD	1.05			1.12			0.98								
*t*^a^	0.14 (2.44)			0.88 (2.44)			0.75 (2.44)								
*F*^a^	1.09 (6.94)			1.86 (6.94)			2.40 (5.78)								

^a^The figures between parenthesis are the tabulated *t* and *F* values, respectively are at *P* = *0.05* [81]

^b^Each result was the average of three separate determinations

**Table 10:** Assay results for the determination of DRT, CAFF, and PAR in synthetic mixtures using derivative method

Mix. No	Ratio	Amount taken (µg/mL)			Amount found (µg/mL)			Percentage found^b^		
		DRT	PAR	CAFF	DRT	PAR	CAFF	DRT	PAR	CAFF
1	1:1.5:10	2.00	3.00	20.00	2.019	3.006	19.887	100.95	100.20	99.44
2	1:1.5:10	3.00	4.50	30.00	2.964	4.472	30.23	98.8	99.38	100.77
3	1:1.5:10	4.00	6.00	40.00	4.017	6.001	39.884	100.43	100.00	99.71
Mean								100.06	99.86	99.97
± S.D								1.12	0.43	0.7
%RSD								1.12	0.43	0.7
%Error								0.65	0.25	0.41
*t*^a^								0.15	0.42	0.63
*F*^a^								1.05	4.59	12.55

^a^The tabulated* t* and *F* values are 2.44, 6.94, respectively at P = 0.05 [81]

^b^Each result was the average of three separate determinations

**Table 11:** Assay results for the determination of DRT, CAFF, and PAR in synthetic mixtures using double divisor method

Mix. No	Ratio	Amount taken (µg/mL)			Amount found (µg/mL)			Percentage found^b^		
		DRT	CAFF	PAR	DRT	CAFF	PAR	DRT	CAFF	PAR
1	1:1.5:10	2.00	3.00	20.00	2.029	1.993	20.069	101.45	100.63	100.35
2	1:1.5:10	3.00	4.50	30.00	2.961	3.017	29.795	98.7	99.20	99.32
3	1:1.5:10	4.00	6.00	40.00	4.024	3.99	40.049	100.6	100.35	100.12
Mean								100.25	100.06	99.93
± S.D								1.41	0.76	0.54
%RSD								1.41	0.76	0.54
%Error								0.81	0.44	0.31
*t*^a^								0.31	0.53	0.70
*F*^a^								1.5	3.95	7.85

^a^The tabulated* t* and *F* values are 2.44, 6.94, respectively at P = 0.05 [81]

^b^Each result was the average of three separate determinations

The selectivity of the method was assessed by observing any interference encountered from the tablet additives cited in the information pamphlet of the studied pharmaceutical preparation (Petro^®^ tablets). About 0.657 g, which approximately equals the weight of one tablet of each additive including magnesium stearate, lactose monohydrate, maize starch, calcium hydrogen phosphate dihydrate, and talc was analyzed using the same procedure described for the analysis of tablets. No interference was encountered from any tablet additive, which confirms the adequate selectivity of the developed method.

#### Applications

##### Application to synthetic mixtures

**Fig. 17:**
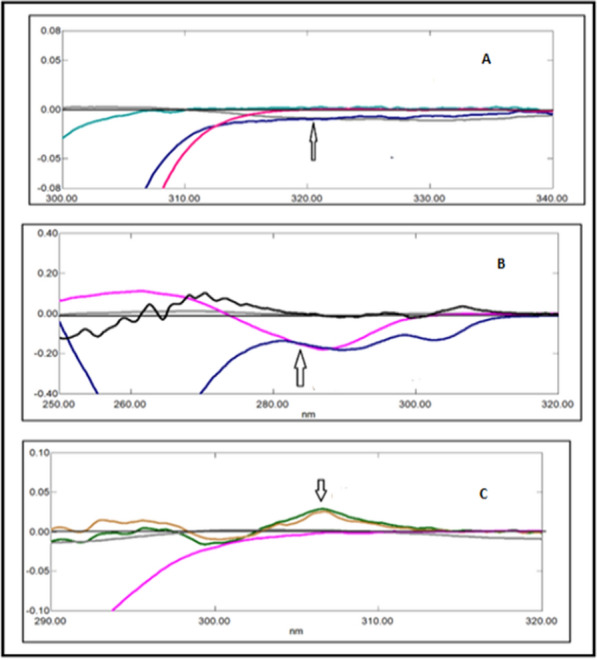
DRT, CAFF and PAR with their synthetic mixtures in derivative method: (**A**) 2 µg/mL DRT with the synthetic mixture containing 2 µg/mL DRT, (**B**) 3 µg/mL CAFF with the synthetic mixture containing 3 µg/mL CAFF, (**C**) 30 µg/mL PAR with the synthetic mixture containing 30 µg/mL PAR

**Fig. 18:**
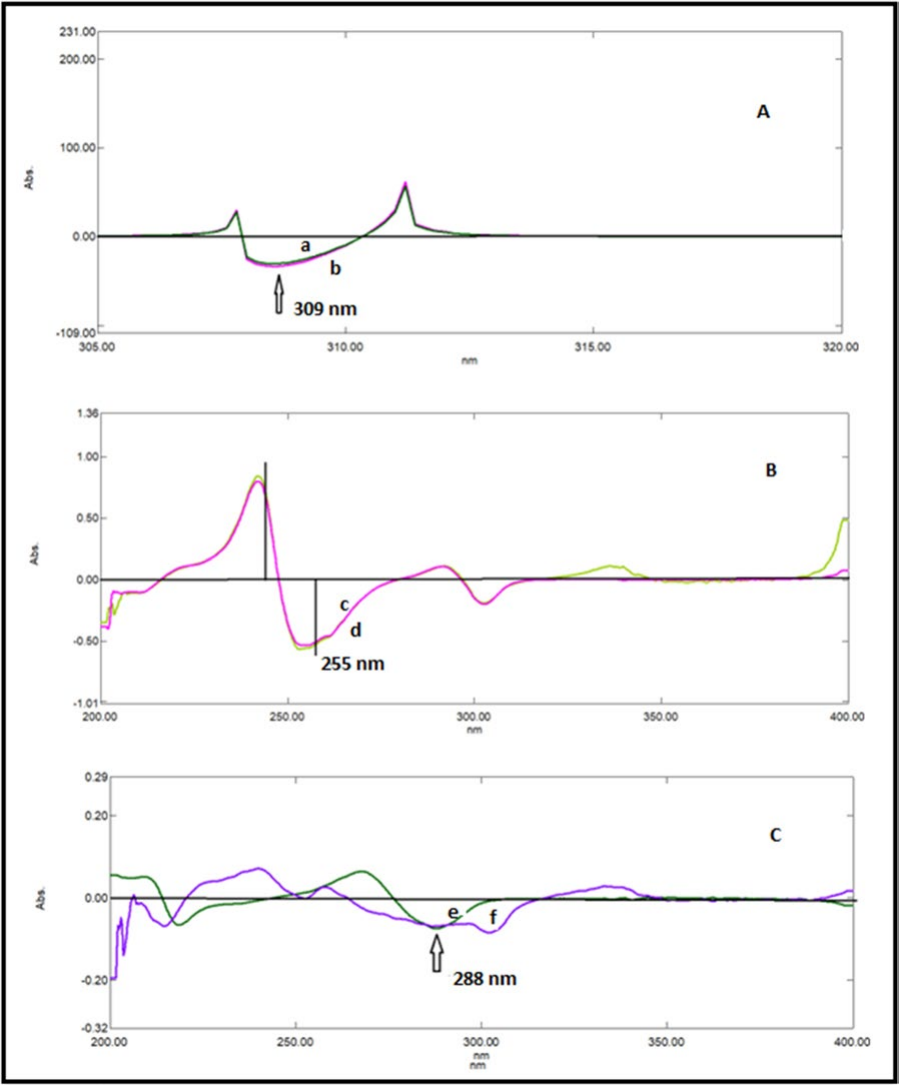
DRT, CAFF and PAR with their synthetic mixtures in double divisor method: (**A**) a is 4 µg/mL DRT with b is the synthetic mixture containing 4 µg/mL DRT, (**B**) c is 40 µg/mL PAR with d is the synthetic mixture containing 40 µg/mL PAR, (**C**) e is 4.5 µg/mL CAFF with f is the synthetic mixture containing 4.5 µg/mL CAFF

The proposed methods utilized to analyze the three drugs in their 1:1.5:10 synthetic mixture in Tables 10 and 11 showed acceptable percentage recoveries for both drugs illustrated in Figs. 17 and 18 in the derivative and double divisor method, respectively.

##### Applications to pharmaceutical formulations

These spectrophotometric methods were excellent applied on the pure bulk powder and on the pharmaceutical preparation: Petro^®^, which contains 40 mg DRT, 60 mg of CAFF, and 400 mg PAR with good accuracy and precision. There was no significant difference after comparing the proposed method with the published method [79] after calculating the student’s t-test and F-value [81] provided in Tables 12, 13, and 14.

**Table 12:** Determination of DRT, CAFF, and PAR in pharmaceutical preparations using the derivative method

Preparation	Proposed method									Comparison method [79]						
	DRT			CAFF			PAR			DRT		CAFF		PAR		
	Amount taken (μg/mL	Amount taken (μg/mL	% Found^b^	Amount taken (μg/mL	Amount taken (μg/mL	% Found^b^	Amount taken (μg/mL	Amount found (μg/mL)	% Found^b^	Amount taken (μg/mL)	%Found^b^	Amount taken (μg/mL)	%Found^b^	Amount taken (μg/mL)		% Found^b^
Petro^®^ tablets (40.0 mg DRT + 60 mg CAFF + 400 mg PAR)	2.00	1.974	98.70	3.00	2.974	99.13	20.00	19.867	99.34	6.00	100.65	6.00	98.97	15.00		98.93
	3.00	3.036	101.20	4.50	4.557	101.27	30.00	30.2	100.67	8.00	98.60	8.00	98.59	20.00		101.90
	4.00	3.977	99.43	6.00	5.975	99.58	40.00	39.846	99.62	10.00	100.52	10.00	101.17	30.00		100.93
‾x ± SD			99.77 ± 1.28			99.99 ± 1.13			99.87 ± 0.7							
*t*^a^			0.15			0.40			0.74							
*F*^a^			1.25			1.52			4.66							

^a^The tabulated* t* and *F* values are *2.44, 6.94*, respectively at *P* = *0.05* [81]

^b^Each result was the average of three separate determination

**Table 13:** Determination of DRT, CAFF, and PAR in pharmaceutical preparations using the double divisor

Preparation	Proposed method									Comparison method [79]					
	CAFF			DRT			PAR			DRT		CAFF		PAR	
	Amount taken μg/mL	Amount found μg/mL	% Found^b^	Amount taken μg/mL	Amount found μg/mL	% Found^b^	Amount taken μg/mL	Amount found μg/mL	% Found^b^	Amount taken μg/mL	% Found^b^	Amount taken μg/mL	% Found^b^	Amount taken μg/mL	% Found^b^
Petro^®^ tablets (40.0 mg DRT + 60 mg CAFF + 400 mg PAR)	2.00	1.978	98.9	3.00	3.006	100.20	20.00	19.817	99.09	6.00	100.65	6.00	98.97	15.00	98.93
	3.00	3.044	101.47	4.50	4.472	99.38	30.00	30.362	101.21	8.00	98.60	8.00	98.59	20.00	101.90
	4.00	3.978	99.45	6.00	6.00	100.00	40.00	39.817	99.54	10.00	100.52	10.00	101.17	30.00	100.93
‾x ± SD			99.94 ± 1.35			99.46 ± 0.43			99.94 ± 1.12						
*t*^a^			0.02			0.33			0.58						
*F*^a^			1.39			10.62			1.84						

^a^The tabulated* t* and *F* values are *2.44, 6.94*, respectively at *P* = *0.05* [81]

^b^Each result was the average of three separate determination

**Table 14:** Determination of DRT, CAFF and PAR in pharmaceutical preparations using the mean centering method

Preparation	Proposed method									Comparison method [79]					
	DRT			CAFF			PAR			DRT		CAFF		PAR	
	Amount taken (μg/mL)	Amount found (μg/mL	% Found^b^	Amount taken (μg/mL	Amount found (μg/mL	% Found^b^	Amount taken (μg/mL	Amount found (μg/mL)	% Found^b^	Amount taken (μg/mL)	% Found^b^	Amount taken (μg/mL)	% Found^b^	Amount taken (μg/mL)	% Found^b^
Petro^®^ tablets (40.0 mg DRT + 60 mg CAFF + 400 mg PAR)	2.00	2.016	100.8	3.00	3.013	100.43	20.00	20.175	100.88	6.00	100.65	6.00	98.97	15.00	98.93
	3.00	2.962	98.73	4.50	4.473	99.40	30.00	29.603	98.68	8.00	98.60	8.00	98.59	20.00	101.90
	4.00	4.022	100.55	6.00	6.013	100.22	40.00	40.213	100.53	10.00	100.52	10.00	101.17	30.00	100.93
‾x ± SD			100.03 ± 1.13			100.02 ± 0.54			100.03 ± 1.18						
*t*^a^			0.11			0.50			0.5						
*F*^a^			1.03			6.55			1.64						

^a^The tabulated* t* and *F* values are *2.44, 6.94*, respectively at *P* = *0.05* [81]

^b^Each result was the average of three separate determination

##### Greenness assessment

Due to the considerable usage of organic solvents in analytical processes, going green can be very difficult. The greenness of these methods was assessed in three different ways.Firstly, Green analytical procedure index (GAPI) [82] The green profiles for the proposed spectrofluorometric methods using the GAPI tool are presented in (Table 15). The 5th parameter was shaded yellow as there was a bit of sample preparation as filtration. Field No. 15 in all techniques had red coloring because there was no waste treatment and the amount of waste was between 1 and 10 mL, thus it was tinted yellow.

**Table 15:** Results for the evaluation of the greenness of the developed spectrophotometric methods by the three green chemistry tools

1. Green analytical procedure index (GAPI)			
			

2- Analytical Eco scale score			
Reagent, volume (mL)	No of Pictograms	Word sign	Penalty points
Reagents / instruments			
Ethanol	2	Danger	4
Item			
Spectrofluorometer	< 0.1 k w h per sample		0
Waste	No treatment		3
Occupational hazards	Analytical process hermitization		0
Total penalty points			$\mathbb{\Sigma}$ 7
Analytical eco scale score			100–7 = 93

3. NEMI pictogram			
			

Analytical eco scale is another quantitative assessment tool Van-Aken et al. [83] published. The proposed methods scored 93, as shown in Table 15. This method is excellent regarding the analytical eco-scale criteria. The penalty points were calculated by the national fire protection association (NFPA) [84].

The National Environmental Method Index (NEMI), an outdated qualitative tool, is the final one [85]. It describes the greenness through a pictogram divided into four quadrants (Table 15). All four quadrants are green colored as no reagents or chemicals are used except ethanol, an eco-friendly solvent. The created approach works well with the three green analytical chemistry tools, which explains why these procedures are environmentally benign, straightforward, quick, and sensitive.

##### In-vitro dissolution test

**Fig. 19:**
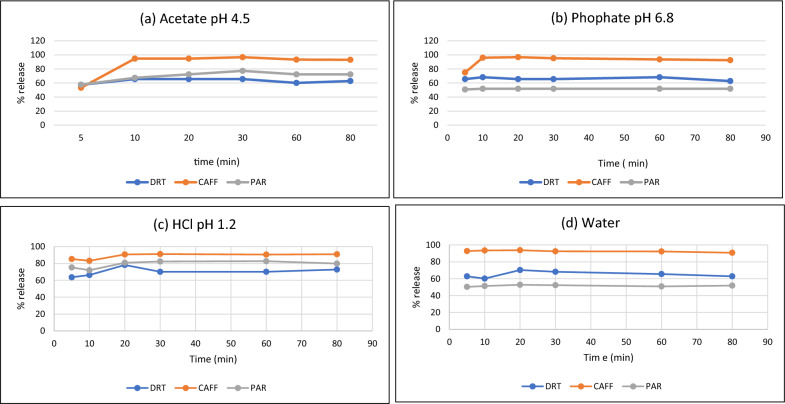
Different dissolution profiles of Petro^®^ tablets in different dissolution media in which: (**a**) Acetate buffer pH 4.5. (**b**) Phosphate buffer pH 6.8. (**c**) 0.1N HCl pH 1.2. (**d**) Water

Dissolution testing has become an essential tool in the pharmaceutical industry at various stages of development, manufacturing and marketing. The in-vitro dissolution profile of Petro^®^ tablets was performed using paddle method. The dissolution media are: HCl solution of pH 1.2, acetate buffer of pH 4.5, phosphate buffer of 6.8 and water [8]. The tablets were placed in 800 ml of medium at 37 °C with stirring speed of 75 rpm. Then, 1 ml of samples were withdrawn out at five, ten, twenty, thirty, sixty and ninety min, and same volume of medium was supplemented to maintain constant medium volume. After filtration using 0.22 μm syringe filters, the samples were analyzed adopting the proposed derivative method according to the procedure 2.4.2. Calibration graphs development. It was found that the release of DRT, CAFF and PAR from its tablets depended on the pH Fig. 19.

## Conclusion

A rapid and simple spectrophotometric approach was devised to simultaneously determine DRT, CAFF, and PAR in response to the demands of quality control laboratories. This straightforward, inexpensive method may be preferable to more expensive, sophisticated ones for routine examination of the examined medications in the co-formulated dosage form.

## Data Availability

All data generated or analysed during this study are included in this published article.
